# Tofacitinib for recurrence of antimelanoma differentiation-associated gene 5 antibody-positive clinically amyopathic dermatomyositis after remission: A case report

**DOI:** 10.1097/MD.0000000000021943

**Published:** 2020-09-11

**Authors:** Yuichi Ishikawa, Tadamichi Kasuya, Michio Fujiwara, Yasuhiko Kita

**Affiliations:** Department of Rheumatology, Yokohama Rosai Hospital, Kohoku-ku, Yokohama, Kanagawa, Japan.

**Keywords:** antimelanoma differentiation-associated gene 5 antibody, clinically amyopathic dermatomyositis, corticosteroid, interstitial lung disease, tofacitinib

## Abstract

**Rationale:**

Antimelanoma differentiation-associated gene 5 antibody (anti-MDA5 Ab)-positive clinically amyopathic dermatomyositis (cADM) is frequently complicated with interstitial lung disease (ILD) and has a poor prognosis. Although the short-term prognosis of anti-MDA5 Ab-positive cADM is poor, it has been suggested that the recurrence rate is not higher than that of anti-MDA5 Ab-negative dermatomyositis. Combination therapy with corticosteroids, calcineurin inhibitors, and cyclophosphamide is the gold standard for the remission induction therapy at the onset. Recently, it has been reported that tofacitinib (TOF) could be effective for refractory anti-MDA5 Ab-positive cADM with ILD. Although initial remission induction therapy has been established, therapeutic strategies for relapse cases have not yet been established.

**Patient concerns:**

A 57-year-old woman who was diagnosed with anti-MDA5 Ab-positive cADM complicated with ILD. In October 2016, she was treated with prednisolone (PSL), tacrolimus (TAC), and cyclophosphamide (CY). These treatments were successful, and PSL could be tapered. However, she developed strong nausea and general fatigue as adverse events of CY. In April 2018, PSL was discontinued, and maintenance therapy was given with TAC. In July 2018, Gottron's sign and ILD recurred. Skin lesions on the finger were partially ulcerated and ILD was also worsening. We proposed a remission reinduction therapy including CY. However, she was rejected CY from experience with past adverse event of CY.

**Diagnosis:**

Based on skin lesions and chest computed tomography (CT) findings, the diagnosis was a recurrence of anti-MDA5 Ab-positive cADM with ILD.

**Interventions:**

Treatment by TOF 10 mg and PSL 22.5 mg (0.5 mg/kg equivalent) was introduced in November 2018.

**Outcomes:**

After introducing TOF and PSL, her skin lesions and chest CT findings of ILD gradually improved. Six months after the induction of TOF, the skin ulcer was epithelialized. One year after the introduction of TOF, PSL was decreased to 9 mg, and the disease activity did not re-exacerbate.

**Lessons:**

This case report is the first report suggesting the effectiveness of TOF for recurrent case of anti-MDA5 Ab-positive cADM with ILD. TOF might be an effective therapeutic option for treating recurrent case of anti-MDA5 Ab-positive cADM.

## 1 Introduction

Dermatomyositis (DM) is an inflammatory myositis with characteristic skin rashes, such as heliotrope rash or Gottron's papule. DM with little or no muscle inflammation is known as clinically amyopathic DM (cADM).^[1]^ cADM is known to be frequently complicated with interstitial lung disease (ILD). In particular, antimelanoma differentiation-associated gene 5 antibody (anti-MDA5 Ab)-positive cADM is frequently complicated with rapidly progressive-ILD and has a poor prognosis.^[2]^ Although the short-term prognosis of anti-MDA5 Ab-positive cADM is very poor, it has been suggested that the recurrence rate is not higher than that of anti-MDA5 Ab-negative DM.^[3]^ Combination therapy with corticosteroids (CS), calcineurin inhibitors such as tacrolimus (TAC), or cyclosporine and cyclophosphamide (CY) is the gold standard for the remission induction therapy at the onset.^[2]^ The efficacy of combination therapy with CS and tofacitinib (TOF) has also been reported, and TOF has attracted attention as a useful therapeutic option for cADM-associated ILD.^[4]^ Moreover, it has been reported that TOF could be effective for refractory anti-MDA5 Ab-positive cADM with ILD.^[5]^ Although several treatment options have been considered for initial remission induction therapy, therapeutic strategies for relapse cases have not yet been established because there have been no large studies into the long-term prognosis and relapse rate of patients with anti-MDA5 Ab-positive DM after remission. In this study, we report the case of anti-MDA5 Ab-positive cADM with recurring ILD and skin lesions after 21 months of starting an initial remission induction therapy treated by a combination of CS and TOF.

## 2 Case report

**Figure 1:**
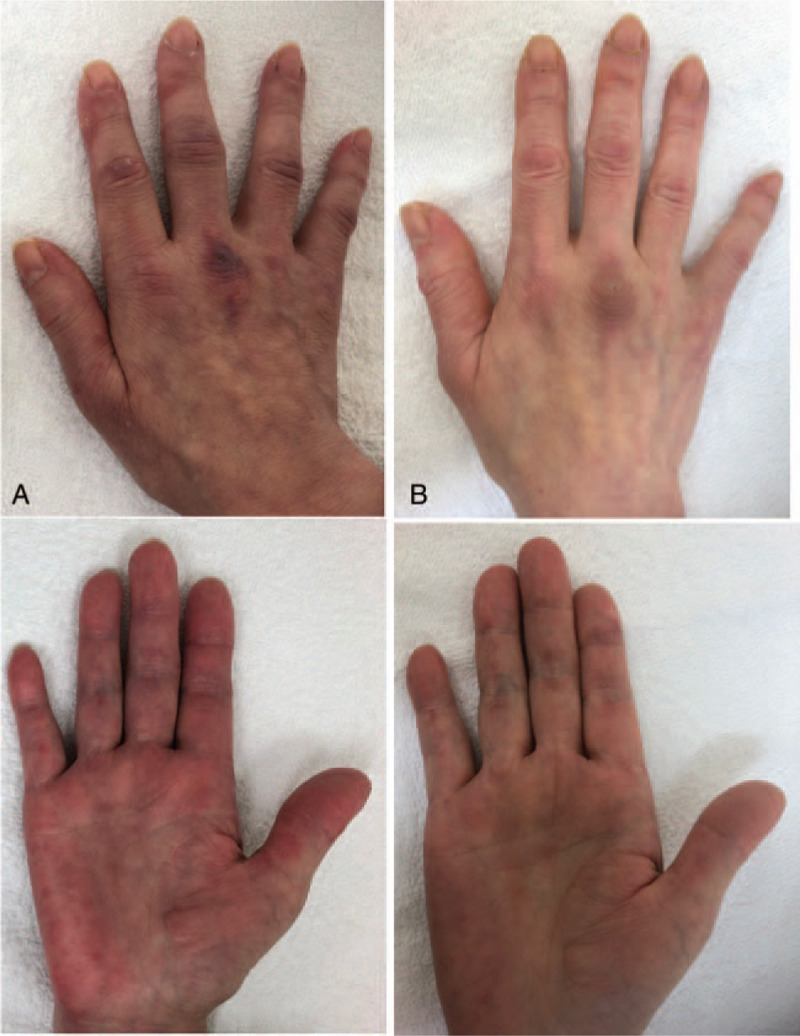
Skin findings. (A) Before the recommencement of prednisolone and introduce of tofacitinib. (B) 6 months after tofacitinib introduction.

**Figure 2:**
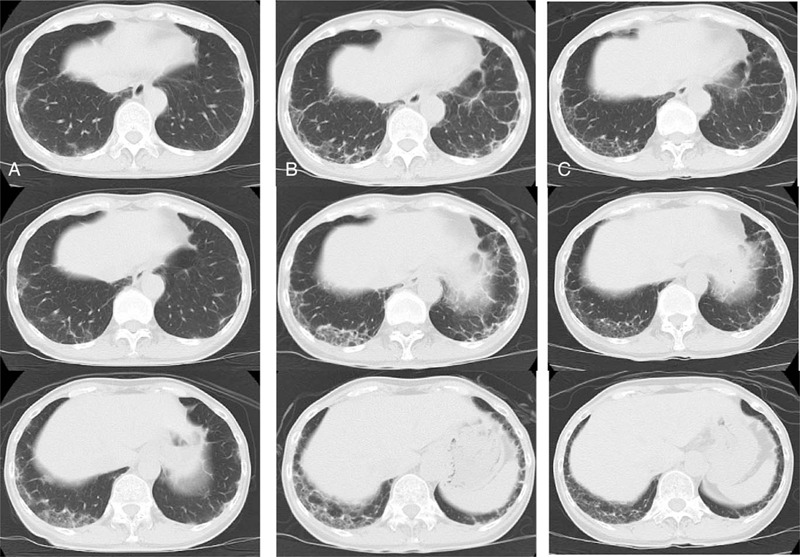
Chest computed tomography. (A) Chest computed tomography (CT) findings on July 2018. (B) Chest CT findings before the recommencement of prednisolone and introduce of tofacitinib (on November 2018). (C) Chest CT findings 6 months after tofacitinib introduction (on May 2019).

**Figure 3:**
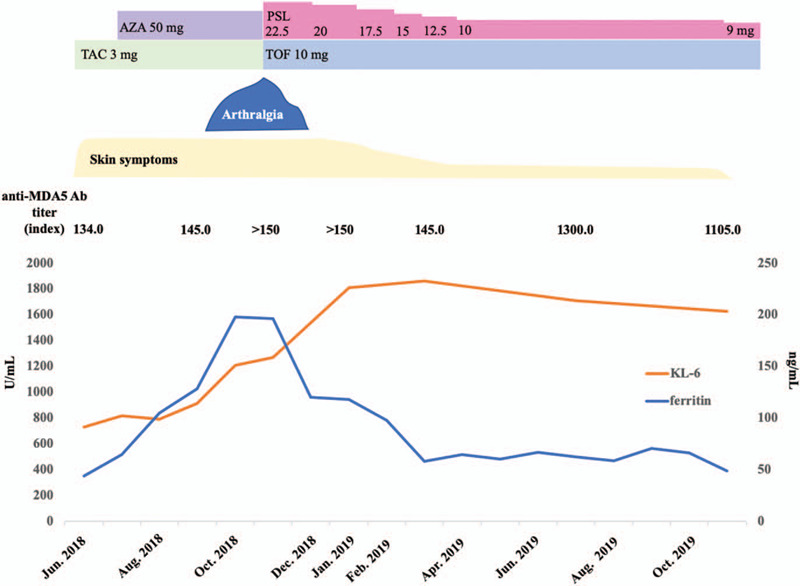
The clinical course of the patient. AZA = azathioprine, KL-6 = Krebs von den Lungen-6, MDA5 Ab = melanoma differentiation-associated gene 5 antibody, PSL = prednisolone, TAC = tacrolimus, TOF = tofacitinib.

A 57-year-old Japanese woman was diagnosed with cADM based on findings such as Gottron's sign and anti-MDA5 Ab-positive status in October 2016. Since her case was complicated with ILD, she was treated with high-dose CS (prednisolone [PSL] 60 mg), TAC 3 mg, and intravenous CY (500 mg/body, administered bi-weekly) as a remission induction therapy. Remission induction therapy was successful: skin lesions and ILD improved. She experienced very strong nausea and general fatigue on CY administration. Because CY was considered an anchor drug for remission induction therapy, we continued to administer CY with an antiemetic. CY was administered 6 times in total, and PSL was gradually tapered with the combination of 3 mg of TAC. In April 2018, PSL could be discontinued, and maintenance therapy was given by TAC. In July 2018, Gottron's sign (Fig. 1A) and ILD relapsed (Fig. 2A). The combined use of azathioprine (AZA) and TAC did not improve disease activity. Skin lesions on the right hand were partially ulcerated. Polyarthritis (both knees and both second to fifth hand metacarpophalangeal joints and proximal interphalangeal joints) also appeared. Since ILD was also getting worse (Fig. 2B), we decided to discontinue immunosuppressants (TAC and AZA) and recommence PSL 22.5 mg (0.5 mg/kg) in November 2018. When the disease activity of cADM recurred (before recommencing PSL), the serum levels of ferritin, LDH, Krebs von den Lungen-6 (KL-6, a surrogate marker of pulmonary fibrosis), and anti-MDA5 Ab titers were elevated (ferritin 197.8 ng/mL, LDH 316 U/mL, KL-6 1271 U/mL, anti-MDA5 Ab > 150 index). Although we considered reintroducing CY as remission reinduction therapy, she refused CY because she had experienced severe adverse events (nausea and general fatigue) with previous CY administration. Based on the previous literature that reported the efficacy of TOF for refractory anti-MDA5 Ab-positive cADM, we decided to introduce TOF 10 mg with PSL (Fig. 3).^[5]^ Two weeks after intensification of the treatment, polyarthritis improved. Six months after the induction of TOF, the skin ulcer was epithelialized (Fig. 1B). Although it took around 6 months for the levels of KL-6 to begin decreasing, the computed tomography (CT) findings of ILD showed an improvement. The lung lesions had not completely improved on chest CT but had progressed without re-exacerbation (Fig. 2C). One year after the introduction of TOF, the dose of PSL was decreased to 9 mg, and the disease activity did not re-exacerbate.

## 3 Discussion

Although the recurrence of anti-MDA5 Ab-positive cADM is said to be relatively infrequent, one study reports a case that relapsed 7 years after the induction of remission.^[6]^ Therefore, it is important to be careful during follow-up even after achieving remission. In the presented case, the recurrence of skin symptoms and ILD was observed during remission maintenance therapy with TAC after discontinuation of PSL. When anti-MDA5 Ab cADM recurred, the serum levels of anti-MDA5 Ab, ferritin, and KL-6 were elevated. Regarding treatment at the time of recurrence, remission reinduction has been reported to be successfully achieved with the same combination therapy of CS, calcineurin inhibitor, and CY as at the time of first remission induction.^[6]^ However, CY can cause adverse events, such as malignancy or gonadal dysfunction, due to the increase in the cumulative amount. Therefore, it is not desirable to administer CY at the time of recurrence in patients with large past doses or those of childbearing age. The efficacy of TOF for anti-MDA5 Ab-positive cADM including refractory cases has been reported previously.^[4,5]^ It has also been suggested that TOF may suppress T cell-derived pro-inflammatory and fibrosis-promoting effects in vitro, and is expected to have a therapeutic effect on intractable ILD associated with cADM.^[7]^ It is suggested that interferon-gamma (IFN-γ) is deeply involved in the etiology of life-threatening DM with ILD, and TOF, which has an effect of suppressing IFN-γ production from T cells, may be effective for refractory DM with ILD.^[8,9]^ In cases such as that presented here, in which readministration of CY is difficult due to adverse events, or in recurrent cases with high levels of pulmonary fibrosing markers such as KL-6, TOF administration might be a useful therapeutic option. Our case was successfully treated without adverse events with a combination of PSL and TOF. Previous reports have shown that the addition of TOF to CS, TAC, or CY has resulted in a large number of adverse events (infectious diseases).^[5]^ If disease activity could be controlled with a combination of CS and TOF, this combination therapy might reduce the incidence of infection as compared with the combination of triple or more immunosuppressants. This is a single case report, and it is necessary to examine the efficacy and safety of this therapy by accumulating cases. The combination therapy of CS and TOF has enabled the control of disease activity, but how long TOF should be administered remains unclear. It is unknown what treatment options should be selected for relapse during TOF administration, and further study is needed to elucidate these points. In conclusion, TOF may be a new effective therapeutic option for recurring anti-MDA5 Ab-positive cADM with ILD cases.

## Author contributions

**Investigation:** Yuichi Ishikawa, Tadamichi Kasuya.

**Supervision:** Michio Fujiwara, Yasuhiko Kita.

**Writing – original draft:** Yuichi Ishikawa.
